# Supplementary material for: Inter- and intra-tumor heterogeneity of metastatic prostate cancer determined by digital spatial gene expression profiling
Source: Nat Commun. 2021 Mar 3;12:1426. doi: 10.1038/s41467-021-21615-4 (PMC7930198; doi:10.1038/s41467-021-21615-4)
Supplement: Supplementary file 1 — Supplementary Information [file 41467_2021_21615_MOESM1_ESM.pdf]

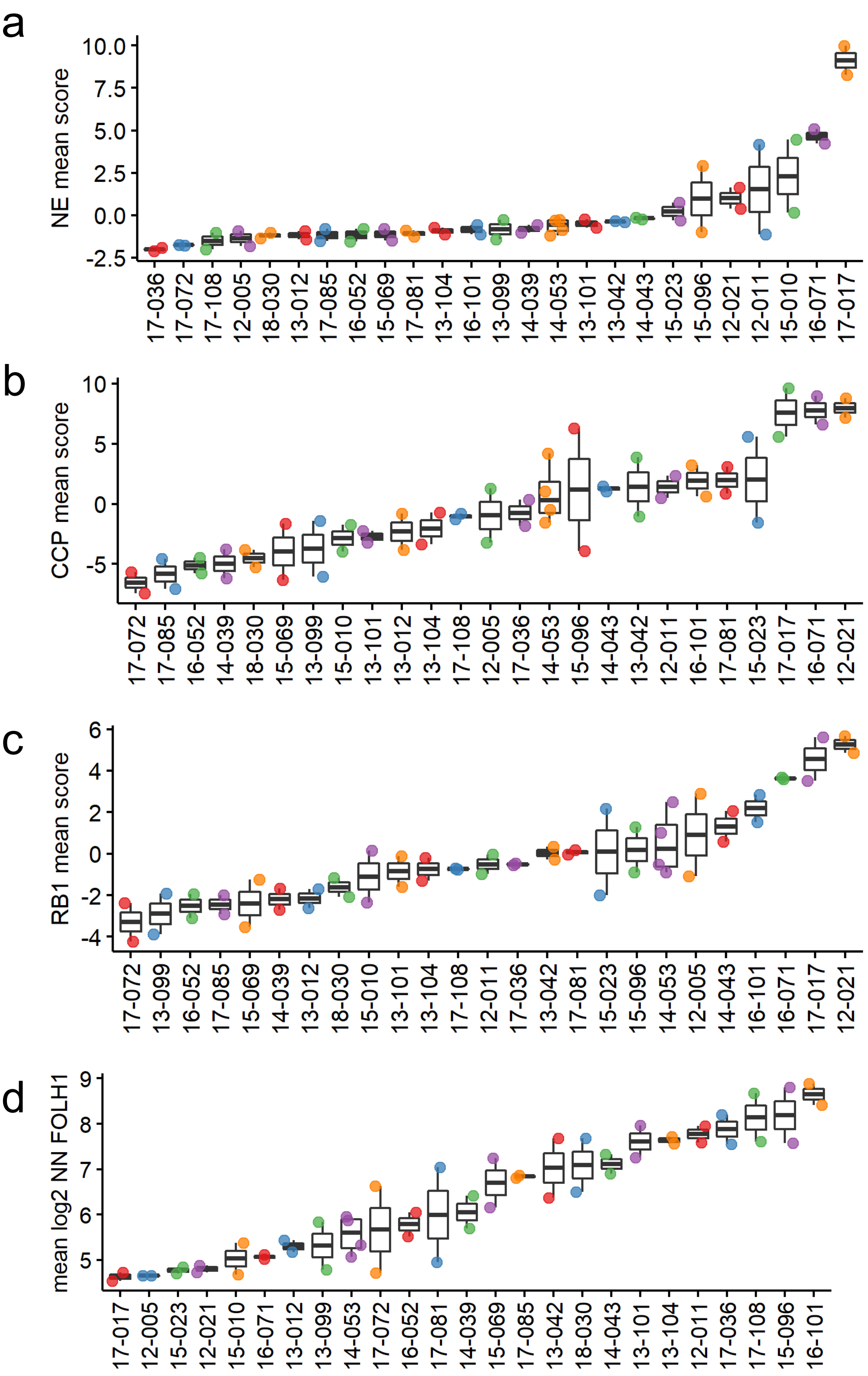

Figure S1. Inter and intra-patient heterogeneity of tumor gene expression determined by DSP. Digital spatial profiling of transcripts from 138 ROIs averaged from 52 tumors from 25 patients with at least two tumors per patient graphed as boxplots demonstrating homogeneous and differential mean expression Z-scores across all patients for: (a) neuroendocrine (NE) activity scores; (b) Cell cycle progression (CCP) scores (c), RB1loss signature scores (d) and log$_2$ negative normalized expression of FOLH1/PSMA. Boxes represent the median and interquartile range (IQR) and the upper and lower whiskers ex-tending to the values that are within 1.5 × IQR; data beyond the end of the whiskers are outliers and plotted as points.

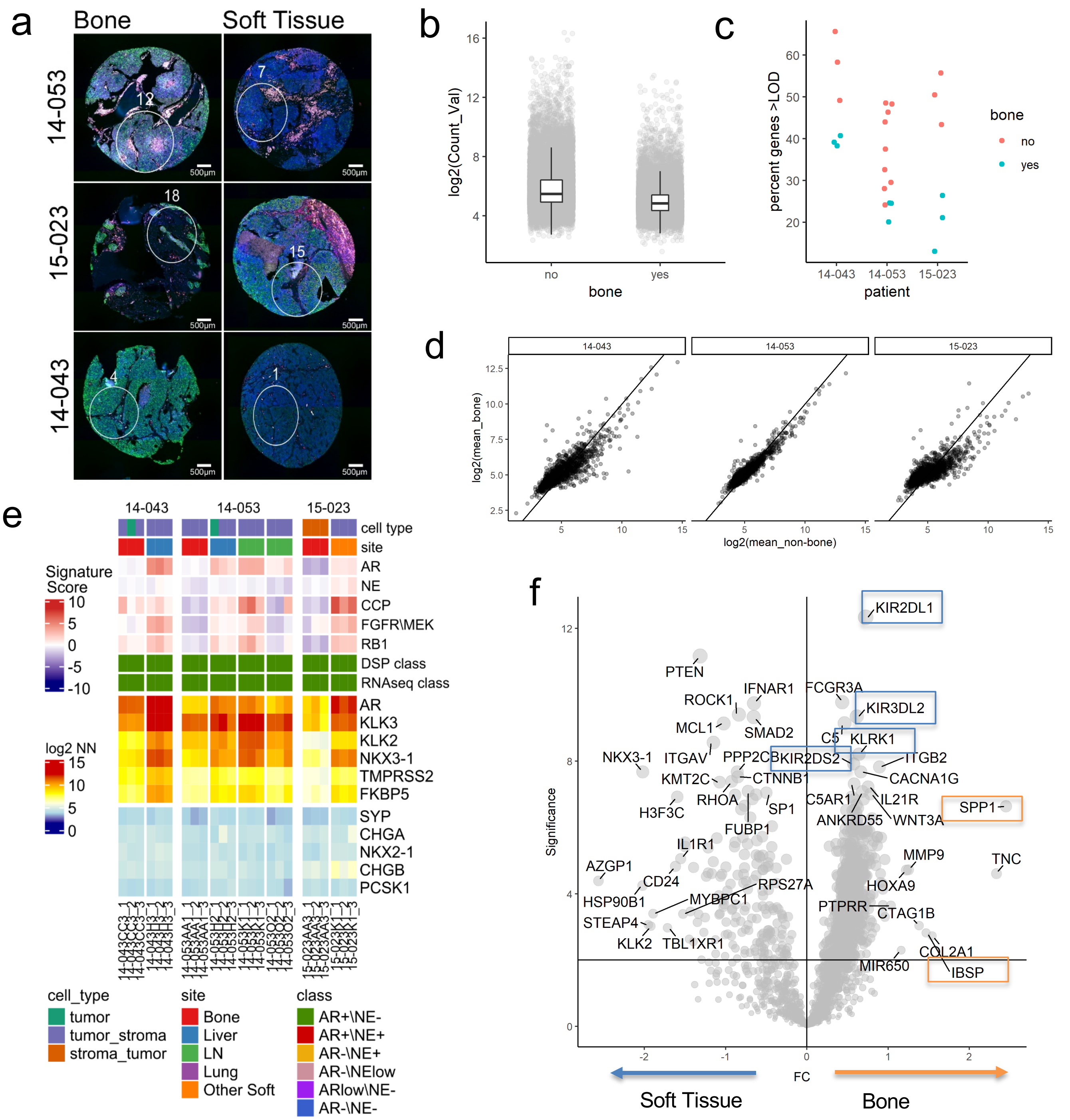

Figure S2. Comparison of gene expression in bone *vs.* soft tissue metastases by DSP.
a. Fluorescent labelling and ROI selection from intra-patient matched bone and soft tissue metastases (N=1 labelling and ROI selection, 1 TMA section).
b-c. Probe counts detected in bone ROIs and percentage of counts above LOD in bone. Boxes represent the median and interquartile range (IQR) and the upper and lower whiskers ex-tending to the values that are within 1.5 × IQR; data beyond the end of the whiskers are outliers and plotted as points.
d. Intra-patient linear regression analyses comparing mean counts between bone and soft tissue metastases ($\log_2$ scale).
e. Heatmap of DSP gene expression comparing bone and soft tissue metastases across AR, NE, CCP, FGFR/MEK, and RB1 gene signatures in 24 ROIs from 3 patients. Results are expressed as gene signature Z-scores and $\log_2$ negative normalized gene expression and presented according to color scales.
f. Volcano plot of soft tissue *vs.* bone metastases. Bone related genes e.g. IBSP and SPP1 are enriched in bone ROIs.

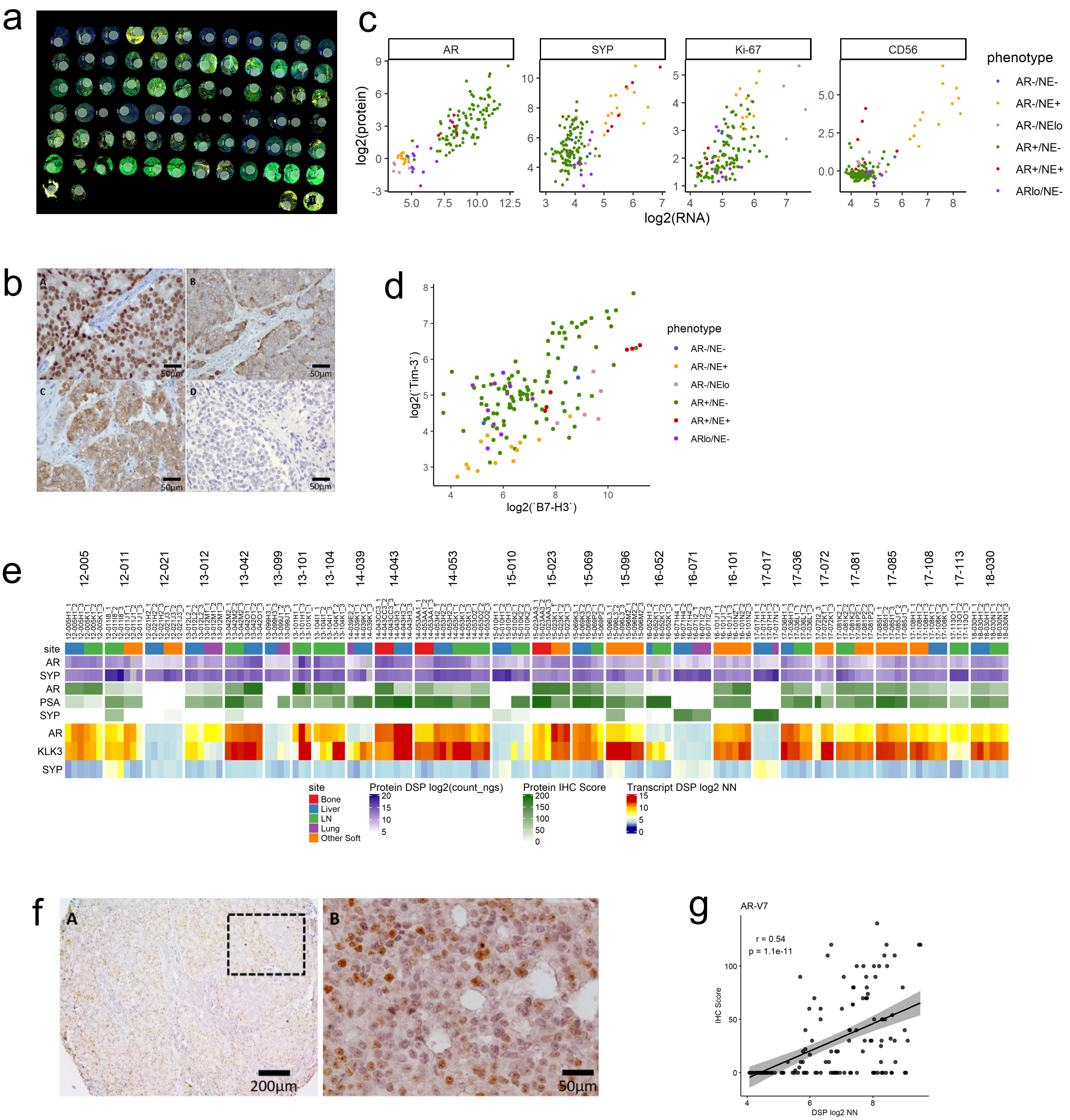

a
b
A
B
C
D
50µm
c
AR
SYP
Ki-67
CD56
log2(protein)
log2(RNA)
phenotype
AR-/NE-
AR-/NE+
AR-/NElo
AR+/NE-
AR+/NE+
ARlo/NE-
d
log2(`Tim-3`)
log2(`B7-H3`)
phenotype
AR-/NE-
AR-/NE+
AR-/NElo
AR+/NE-
AR+/NE+
ARlo/NE-
e
12-005
12-011
12-021
13-012
13-042
13-099
13-101
13-104
14-039
14-043
14-053
15-010
15-023
15-069
15-096
16-052
16-071
16-101
17-017
17-036
17-072
17-081
17-085
17-108
17-113
18-030
site
AR
SYP
AR
PSA
SYP
AR
KLK3
SYP
site
Bone
Liver
LN
Lung
Other Soft
Protein DSP log2(count_ngs)
Protein IHC Score
Transcript DSP log2 NN
f
A
B
200µm
50µm
g
AR-V7
r = 0.54
p = 1.1e-11
IHC Score
DSP log2 NN

Figure S3. DSP protein quantitation and comparisons with orthogonal analysis methods.

a. ROI selection of each fluorescently labelled TMA core. ROIs are super-imposed onto a serial section of the TMA. Slides for protein DSP were stained with PanCK (green), Syto 13 (blue) and CD45 (yellow). (N=1 fluorescent labelling, 1 TMA section).

b. Representative IHC staining. A) positive AR staining, sample 13-101K1, B) positive PSA staining, sample 17-108K1, C) positive SYN staining, sample 16-071 H4 and D) negative control IgG, sample 15-096M1. All images are at 40X magnification N=1 IHC staining, 1 TMA section.

c. Scatterplot comparing protein vs. RNA data from the same ROI. Data is $\log_2$ transformed. Patient samples are grouped by phenotype, and phenotype expression scores compared across individual genes AR, SYP, Ki-67 and CD56 (n=141).

d. Correlation of B7-H3 and TIM-3 protein abundance by DSP protein quantitation (n=138).

e. Heatmap of bulk RNA seq gene expression, IHC and protein DSP for AR, KLK3 (PSA) and SYP of 141 ROIs from 26 patients presented according to color scales.

f. Representative heterogeneous AR-V7 nuclear expression. A) 10X magnification. Dotted lines represent enhanced magnification in image B) (40X). N=1 IHC TMA staining, 1 TMA section.

g. Scatterplot between AR-V7 transcript DSP and IHC Score (n=139 ROIs from 26 patients.) Two-sided test for association using Pearson's correlation coefficient, r; p-value shown on plot. NN – negative normalized.
