# Supplementary material for: Inter- and intra-tumor heterogeneity of metastatic prostate cancer determined by digital spatial gene expression profiling
Source: Nat Commun. 2021 Mar 3;12:1426. doi: 10.1038/s41467-021-21615-4 (PMC7930198; doi:10.1038/s41467-021-21615-4)
Supplement: Supplementary file 3 — Description of Additional Supplementary Files [file 41467_2021_21615_MOESM3_ESM.pdf]

Description of Additional Supplementary Files

File Name: Supplementary Data 1
Description: Genes analyzed by DSP Transcript Profiling

File Name: Supplementary Data 2
Description: Tumor and ROI Information

File Name: Supplementary Data 3
Description: Digital Spatial Profile Transcript Data

File Name: Supplementary Data 4
Description: Digital Spatial Profile Protein Data
