# Supplementary material for: Inter- and intra-tumor heterogeneity of metastatic prostate cancer determined by digital spatial gene expression profiling
Source: Nat Commun. 2021 Mar 3;12:1426. doi: 10.1038/s41467-021-21615-4 (PMC7930198; doi:10.1038/s41467-021-21615-4)
Supplement: Supplementary file 9 — Reporting Summary [file 41467_2021_21615_MOESM9_ESM.pdf]

nature research

Corresponding author(s): Peter S. Nelson

Last updated by author(s): Dec 22, 2020

Reporting Summary

Nature Research wishes to improve the reproducibility of the work that we publish. This form provides structure for consistency and transparency in reporting. For further information on Nature Research policies, see our Editorial Policies and the Editorial Policy Checklist.

Statistics

For all statistical analyses, confirm that the following items are present in the figure legend, table legend, main text, or Methods section.

n/a	Confirmed	
☐	☒	The exact sample size (n) for each experimental group/condition, given as a discrete number and unit of measurement
☐	☒	A statement on whether measurements were taken from distinct samples or whether the same sample was measured repeatedly
☐	☒	The statistical test(s) used AND whether they are one- or two-sided<br>*Only common tests should be described solely by name; describe more complex techniques in the Methods section.*
☒	☐	A description of all covariates tested
☐	☒	A description of any assumptions or corrections, such as tests of normality and adjustment for multiple comparisons
☐	☒	A full description of the statistical parameters including central tendency (e.g. means) or other basic estimates (e.g. regression coefficient) AND variation (e.g. standard deviation) or associated estimates of uncertainty (e.g. confidence intervals)
☐	☒	For null hypothesis testing, the test statistic (e.g. F, t, r) with confidence intervals, effect sizes, degrees of freedom and P value noted<br>*Give P values as exact values whenever suitable.*
☒	☐	For Bayesian analysis, information on the choice of priors and Markov chain Monte Carlo settings
☒	☐	For hierarchical and complex designs, identification of the appropriate level for tests and full reporting of outcomes
☐	☒	Estimates of effect sizes (e.g. Cohen's d, Pearson's r), indicating how they were calculated

Our web collection on statistics for biologists contains articles on many of the points above.

Software and code

Policy information about availability of computer code

Data collection	GSVA (version 1.32.0); STAR alignment (version 2.7.3a); Gene level abundance was quantitated from the filtered human alignments in R using the Genomic Alignments Bioconductor package (version 1.22.1). All analysis in R was performed with R version 3.5.1 or 3.6.2 and RStudio (1.3.1093).
Data analysis	Enrichment scores (ES) were calculated in R using the z-scores function within the GSVA package 74 with default parameters and log2 negative normalized expression values above background as input. All signatures are described in Nyquist et al. 31. Sample phenotypic groups were visualized using classical multidimensional scaling (MDS) calculated with the cmdscale function in R using the expression of 23 out of 26 genes in a published gene signature 75. Three genes (ACTL6B, S100A14 and FGFBP1) were removed due to lack of expression in the DSP dataset. The distance metric was "euclidean" calculated by dist function on the columns (samples).

For manuscripts utilizing custom algorithms or software that are central to the research but not yet described in published literature, software must be made available to editors and reviewers. We strongly encourage code deposition in a community repository (e.g. GitHub). See the Nature Research guidelines for submitting code & software for further information.

Data

Policy information about availability of data

All manuscripts must include a data availability statement. This statement should provide the following information, where applicable:

- Accession codes, unique identifiers, or web links for publicly available datasets
- A list of figures that have associated raw data
- A description of any restrictions on data availability

All of the relevant data for this study are publicly available and have been provided by the authors. The RNAseq data used in this study are available under GEO

accession number GSE147250 [https://www.ncbi.nlm.nih.gov/geo/query/acc.cgi?acc=GSE147250]. The DSP transcript data are provided in Supplementary Data File 3. The DSP protein data are provided in Supplementary Data File 4.

Field-specific reporting

Please select the one below that is the best fit for your research. If you are not sure, read the appropriate sections before making your selection.

- [x] Life sciences
- [ ] Behavioural & social sciences
- [ ] Ecological, evolutionary & environmental sciences

For a reference copy of the document with all sections, see nature.com/documents/nr-reporting-summary-flat.pdf

Life sciences study design

All studies must disclose on these points even when the disclosure is negative.

Sample size	No prespecified samples sizes were determined. The sample size resulted from profiling/analyzing all metastatic samples available to the research team
Data exclusions	Several biospecimens/TMA cores were excluded due to: Manual inspection of the TMAs used for DSP determined that of 168 cores arrayed, 7 were either missing or were 100% fat and one was entirely stroma devoid of tumor cells. For the RNA DSP assay, 7 additional ROIs were missing and 4 did not pass sequencing quality control. These cores were excluded from further analyses for both protein and RNA experiments.
Replication	Orthogonal assays were used to assess concordance for gene expression. These included DSP transcript, DSP protein, bulk RNAseq and immunohistochemistry. DSP transcript, DSP protein and immunohistochemistry were performed with n=1 FFPE tissuemicroarray sections, with three spatially distinct cores per tumor included. Bulk RNA-seq was performed with 1ug of total RNA isolated from each tumor.
Randomization	There were no experimental groups. All samples available to analyzed using similar approaches and subsequently partitioned into groups based on gene expression.
Blinding	Experiments performed in this study include DSP transcript, DSP protein, bulk RNA-seq and immunohistochemistry. The investigators performing DSP and bulk RNA seq and IHC were blinded as to the results of the orthogonal methods.

Reporting for specific materials, systems and methods

We require information from authors about some types of materials, experimental systems and methods used in many studies. Here, indicate whether each material, system or method listed is relevant to your study. If you are not sure if a list item applies to your research, read the appropriate section before selecting a response.

Materials & experimental systems

n/a	Involved in the study	
[ ]	[x]	Antibodies
[x]	[ ]	Eukaryotic cell lines
[x]	[ ]	Palaeontology and archaeology
[x]	[ ]	Animals and other organisms
[ ]	[x]	Human research participants
[x]	[ ]	Clinical data
[x]	[ ]	Dual use research of concern

Methods

n/a	Involved in the study	
[x]	[ ]	ChIP-seq
[x]	[ ]	Flow cytometry
[x]	[ ]	MRI-based neuroimaging

Antibodies

Antibodies used

For individual antibodies used for immunohistochemistry: Tissue sections were blocked with 5% normal goat-horse-chicken serum, incubated with primary antibody anti-Androgen Receptor Biogenex MU256-UC (1:60), anti-Androgen Receptor V7 anti-gen (clone RM7) RevMab Biosiences (1:2000), anti-Prostate-specific Antigen Dako A0562 1:1000, anti-Synaptophysin Santa Cruz sc-17750 (1:200), incubated with biotinylated secondary antibody(Vector Laboratories Inc.), followed by ABC reagent (Vector Laboratories Inc.), and stable DAB (Invitrogen Corp.). Mouse and Rabbit IgG antibodies acted as controls and were used at the same concentration as the primary antibodies (Rabbit IgG I-1000-5 Vector Labs, mouse IgG MOPC-21 developed by the Genitourninary Cancer Research Lab at the University of Washington). Fluorescent antibodies were used to identify ROIs of interest, pan-cytokeratin, CD3 and CD45.

For antibody-based Digital Spatial Profiling: Oligonucleotide-conjugated antibodies were used in these studies. Sixty antibodies conjugated to unique PC-oligos were mixed into a single cocktail for these studies. NanoString provides a NanoString Protein Probe ID for each unique antibody conjugation clone in lieu of specific identifying information about the antibodies as NanoString considers this confidential information. Conjugated IHC validation was performed for most antibodies in these studies. Antibody validation was performed on positive-control FFPE tissue samples and FFPE cell pellets. A Table of antibodies used - 57 antibodies for human protein quantification and 3 control antibodies is provided as Supplementary Data File 5.
The antibodies used are:

4-1BB
AR
ARG1
B7-H3
BCL2
B2M
CD11c
CD127
CD14
CD163
CD20
CD25
CD27
CD3
CD34
CD4
CD40
CD44
CD45
CD45RO
CD56
CD66b
CD68
CD8
CD80
CTLA4
EpCAM
ER alpha
FAP alpha
Fibronectin
FOXP3
GAPDH
GITR
GZMB
Her2/ErbB2
Histone H3
HLA-DR
ICOS
IDO1
Ki-67
LAG3
MART1
Ms IgG1**
Ms IgG2a**
NY-ESO-1
OX40L
PanCk
PD-1
PD-L1
PD-L2
PR
PTEN
Rb IgG***
S100B
S6
SMA
STING
SYP
Tim-3
VISTA

** anti-mouse; *** anti-rabbit

Validation

Anti-Androgen Receptor - http://store.biogenex.com/us/applications/ihc/controls/controls/anti-androgen-receptor-clone-f39-4-1.html
Anti-Androgen Receptor V7 - https://www.revmab.com/index.php/product/anti-androgen-receptor-ar-v7-specific-rabbit-monoclonal-antibody-clone-rm7-arv7-splice-variant/
Anti-Prostate Specific Antigen - https://www.labome.com/product/Dako/A0562.html
Anti-Synaptophysin - https://www.scbt.com/p/syp-antibody-d-4

Mouse IgG - Developled by the Genitourninary Cancer Research Lab at the University of Washington. MOPC-21 is a non-specific 21 amino acid sequence used to determine nonspecific binding.
Rabbit IgG - https://vectorlabs.com/rabbit-igg.html.
Pan- Cytokeratin - https://www.novusbio.com/products/cytokeratin-pan-antibody-ae-1-ae-3_nbp2-33200
CD3 - https://www.origene.com/catalog/antibodies/primary-antibodies/um500048/cd3e-mouse-monoclonal-antibody-clone-id-umab54
CD45 - https://www.novusbio.com/products/cd45-antibody-2b11-pd7-26_nbp2-34528

Human research participants

Policy information about studies involving human research participants

Population characteristics	All patients in this study were diagnosed with advanced prostate cancer. All patients were male and died of metastatic castration resistant prostate cancer, and subsequently underwent a rapid autopsy procedure for tumor procurement. No additional co-variate information was used for patient inclusion.
Recruitment	All rapid autopsy tissues were collected from patients who signed written informed consent under the aegis of the Prostate Cancer Donor Program at the University of Washington. Every suitable advanced stage patient with terminal disease is considered for informed consent for donating tissues to research, however there is no uniform process as this conversation is delicate and is had with the patient and the family depending upon the judgment of the attending physician. The majority of patients who enroll in the program are patients attending the medical oncology and urological oncology clinics at the University of Washington, however, occasionally patients and their families reach out to the program looking to participate. Furthermore, the patient must be within a two hour radius of the University of Washington at time of death, as the ambulance team have to pick up and return the body to the morgue post haste. This leads to selection bias, as there is small minority community who attend clinics at the University of Washington and live in the Seattle area. Limiting the ability of the program to recruit minority patients.
Ethics oversight	Samples were obtained from patients who died of metastatic castration resistant prostate cancer and who had provided written informed consent as per the aegis of the Prostate Cancer Donor Program at the University of Washington. The Institutional Review Board of the University of Washington approved this study.

Note that full information on the approval of the study protocol must also be provided in the manuscript.
